# Supplementary material for: Spatiotemporal Dynamics of Covert Versus Overt Processing of Happy, Fearful and Sad Facial Expressions
Source: Brain Sci. 2021 Jul 17;11(7):942. doi: 10.3390/brainsci11070942 (PMC8329921; doi:10.3390/brainsci11070942)
Supplement: Supplementary file 1 [file brainsci-11-00942-s001.zip › brainsci-1270522-supplementary.pdf]

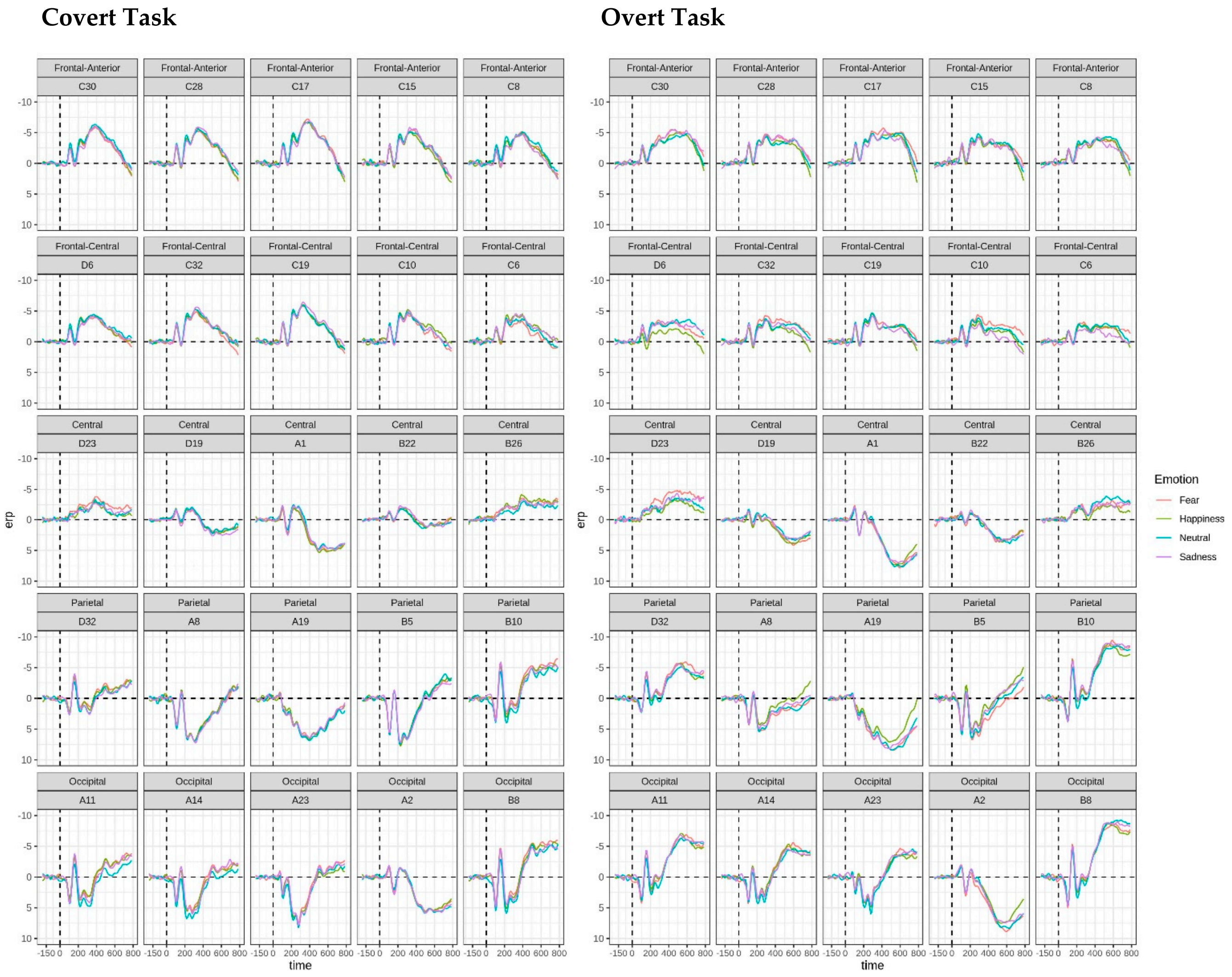

Supplementary Figure S1: ERP grandaverage waveforms to Fearful, Happy, Sad, and Neutral expressions in the Covert task (on left) and the Overt task (on the right) at 25 representative scalp sites ordered top to bottom into frontal anterior, frontocentral, frontal, parietal, and occipital scalp regions, and left to right into left ventral, left dorsal, midline, right dorsal, right ventral. Negative voltage is up.

**Early Window** (140-180 ms)	Negative Cluster			Positive Cluster		
Emotion main effect	Cohen's d	CI Lower	CI Upper	Cohen's d	CI Lower	CI Upper
Happy vs Neutral	1.35	-1.53	-1.06	1.17	0.77	1.17
Sad vs Neutral	1.58	-1.61	-1.18	1.07	0.84	1.33
Happy vs Sad	1.08	-1.31	-0.83	--	--	--
Happy vs Fear	1.37	-1.23	-0.86	--	--	--
**Intermediate Window** (200-400 ms)						

Condition main effect	Cohen’s d	CI Lower	CI Upper	Cohen’s d	CI Lower	CI Upper
Overt vs Covert	2.1	-4.6	-3.65	2.06	2.42	3.06
Emotion main effect						
Emotion vs Neutral	1.2	-1.8	-1.19	--	--	--
**Late Window** (500-800 ms)						
Condition X Emotion	Cohen’s d	CI Lower	CI Upper	Cohen’s d	CI Lower	CI Upper
Fear vs Happy Overt	1.36	-3.01	-2.1	1.79	4.02	5.27
Sad vs Happy Overt	--	--	--	1.39	1.96	2.78
Happy vs Neutr Overt	1.05	-2.07	-1.29	--	--	--

Supplementary Table S1- Effect size (Cohen’s d) and lower and upper confidence intervals for the significant post-hoc pairwise mass-univariate tests showing significant negative and positive clusters. Top: Early time window (140-180 ms), main effect of Emotion. Center: Intermediate time window (200-400 ms), main effects of Condition (above) and Emotion (below). Bottom: Late time window (500-800 ms), Condition X Emotion interaction (only the contrasts discussed in the paper).

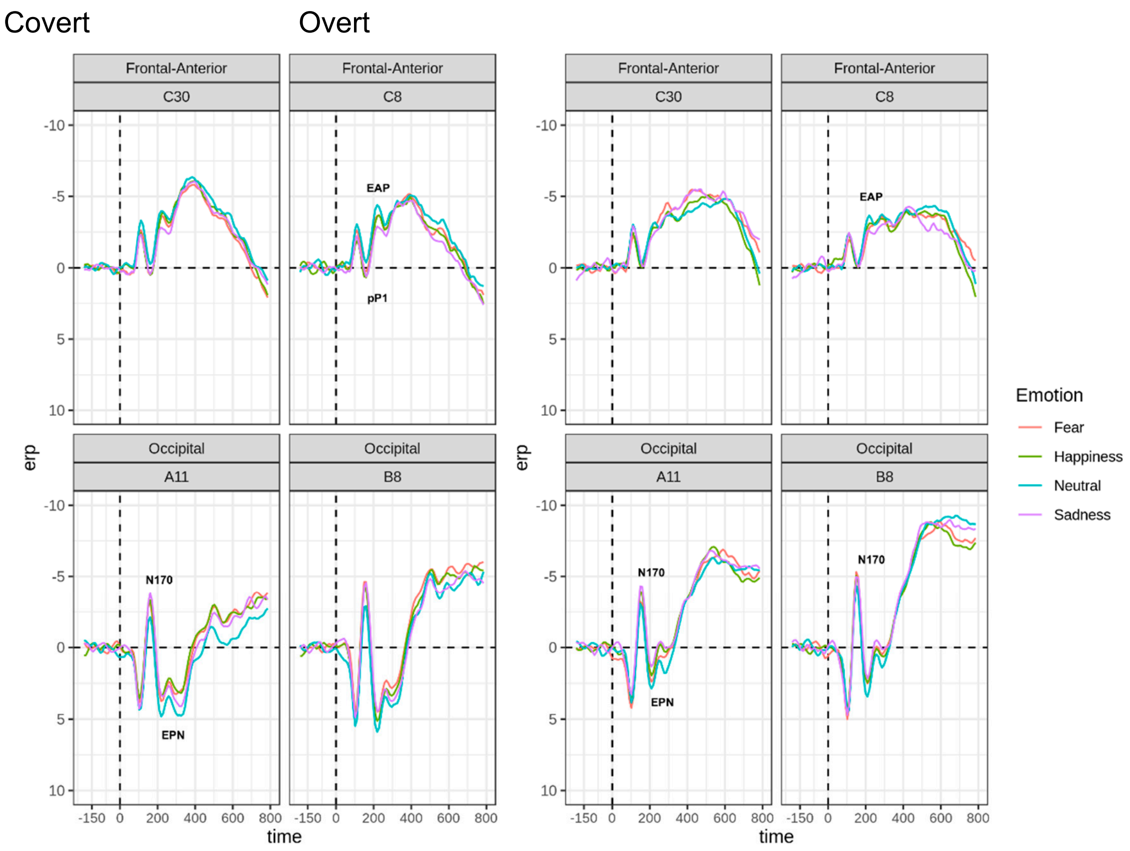

Supplementary Figure S2: ERP grandaverage waveforms to Fearful, Happy, Sad, and Neutral expressions in the Covert task (on left) and the Overt task (on the right) at the left and right occipital

and anterior frontal sites showing the largest emotion modulations for the N170 and the EPN and their frontal counterparts. Negative voltage is up.

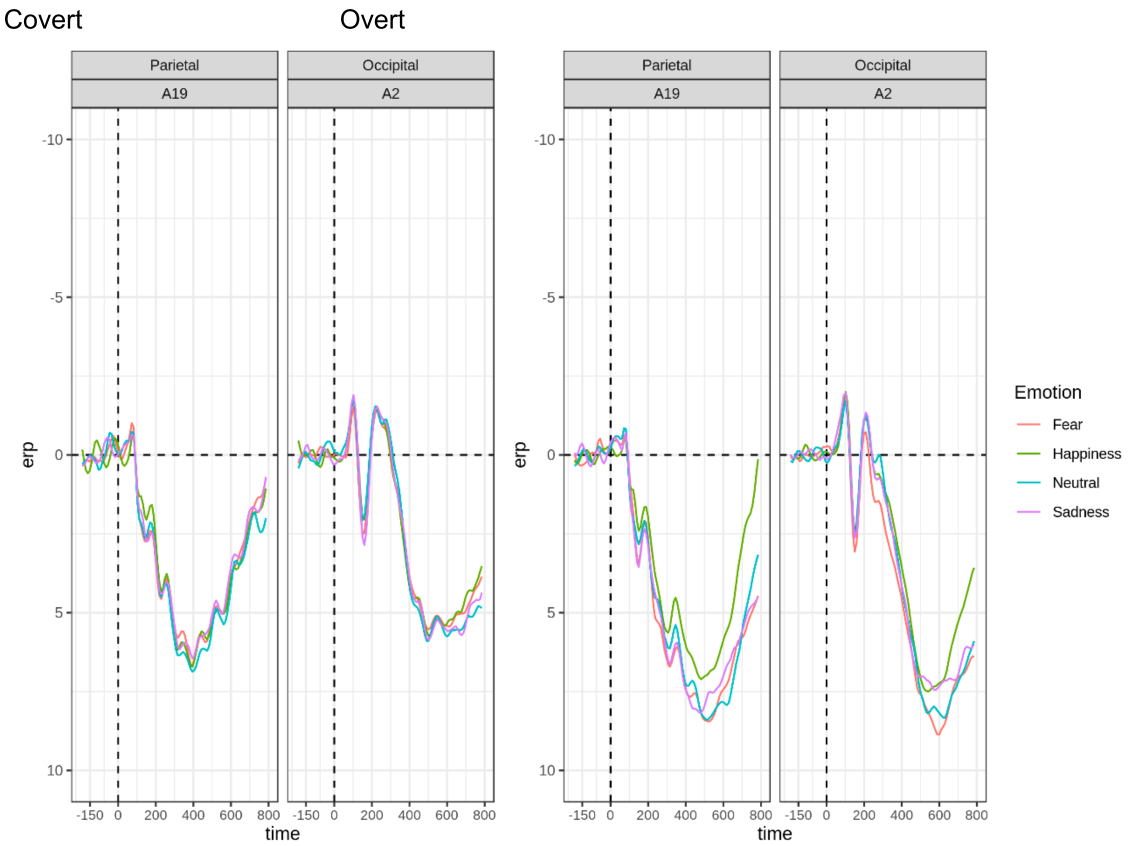

Supplementary Figure S3: ERP grandaverage waveforms to Fearful, Happy, Sad, and Neutral expressions in the Covert task (on left) and the Overt task (on the right) at midline parietal and occipital sites showing the largest emotion modulations for the LPP. Negative voltage is up.
